# Masked Speech Recognition in School-Age Children

**DOI:** 10.3389/fpsyg.2019.01981

**Published:** 2019-09-03

**Authors:** Lori J. Leibold, Emily Buss

**Affiliations:** ^1^Human Auditory Development Laboratory, Department of Research, Center for Hearing Research, Boys Town National Research Hospital, Omaha, NE, United States; ^2^Psychoacoustics Laboratories, Department of Otolaryngology/Head and Neck Surgery, University of North Carolina at Chapel Hill, Chapel Hill, NC, United States

**Keywords:** development, children, hearing, speech perception, masking

## Abstract

Children who are typically developing often struggle to hear and understand speech in the presence of competing background sounds, particularly when the background sounds are also speech. For example, in many cases, young school-age children require an additional 5- to 10-dB signal-to-noise ratio relative to adults to achieve the same word or sentence recognition performance in the presence of two streams of competing speech. Moreover, adult-like performance is not observed until adolescence. Despite ample converging evidence that children are more susceptible to auditory masking than adults, the field lacks a comprehensive model that accounts for the development of masked speech recognition. This review provides a synthesis of the literature on the typical development of masked speech recognition. Age-related changes in the ability to recognize phonemes, words, or sentences in the presence of competing background sounds will be discussed by considering (1) how masking sounds influence the sensory encoding of target speech; (2) differences in the time course of development for speech-in-noise versus speech-in-speech recognition; and (3) the central auditory and cognitive processes required to separate and attend to target speech when multiple people are speaking at the same time.

## Introduction

Children must learn how to communicate in noisy environments such as classrooms (e.g., Knecht et al., 2002). Thus, it is not surprising that extensive research conducted over the past 30 years has focused on understanding children’s masked speech recognition abilities (e.g., Elliott, 1979; Hall et al., 2002; Brown et al., 2010; McCreery et al., 2017; Dillon et al., 2018). Several consistent trends have emerged from this research. First, the detrimental effects of auditory masking on speech recognition are larger for children than for adults (reviewed by Erickson and Newman, 2017). Second, the ability to recognize speech in the presence of competing sounds develops throughout the school-age years and does not mature until adolescence (e.g., Cameron et al., 2009; Brown et al., 2010; Corbin et al., 2016). Finally, children’s increased susceptibility to auditory masking relative to adults in the context of speech recognition is more pronounced and prolonged when the masker is also speech than when the masker is steady-state noise (e.g., Hall et al., 2002; Corbin et al., 2016). These results have collectively had significant impact on public health policy, leading to the establishment of classroom standards for noise levels (ANSI, 2010) as well as recommendations that speech-in-noise testing be included in the pediatric audiology test battery.

While children’s considerable masked speech recognition difficulties have been well documented, a comprehensive model of the factors responsible for developmental effects has not been established. This review aims to characterize child/adult differences in the ubiquitous problem of recognizing speech in the presence of competing background sounds, with a specific goal of summarizing the literature pertaining to factors thought to be responsible for age-related changes in performance. The review begins with an overview of children’s speech recognition abilities in steady-state noise. Historically, the development of speech-in-noise recognition has been a major focus for researchers in the field. This focus partly reflects an early emphasis on understanding bottom-up contributions to development, based on the premise that speech recognition in steady-state noise requires an accurate sensory representation of target speech. Findings from studies investigating the influence of top-down contributions of language knowledge and cognitive processing on children’s recognition of speech that has been degraded by noise are then discussed. Building on this foundational work, the latter half of the review concentrates on age effects on the ability to recognize speech when several people are talking in the background. The research summarized in this section provides compelling evidence that central auditory and cognitive processing play a critical role in the development of speech-in-speech recognition. Finally, areas for future research are briefly highlighted.

## Speech-in-Noise Recognition

Children are poorer than adults are at recognizing phonemes, words, or sentences in a background of steady-state noise (e.g., Elliott, 1979; Nittrouer and Boothroyd, 1990; McCreery and Stelmachowicz, 2011; Dillon et al., 2018). For example, McCreery and Stelmachowicz (2011) evaluated syllable recognition in a speech-shaped noise masker. Participants were a large sample of 5- to 12-year-old children (*n* = 116) and young adults with normal hearing. Stimulus bandwidth was manipulated *via* filtering, and testing was completed at multiple signal-to-noise ratios (SNRs). Children consistently required more favorable SNRs than adults to achieve comparable performance. Similar child/adult differences have been reported using word and sentence stimuli (e.g., Buss et al., 2017), and findings from related studies indicate that children require greater spectral detail relative to adults in order to recognize filtered speech (Eisenberg et al., 2000; Mlot et al., 2010).

A closer examination of the literature reveals that speech-in-noise recognition improves gradually over the first decade of life; adult-like performance is not usually observed until 9–10 years when stimuli are presented diotically (e.g., Eisenberg et al., 2000; Corbin et al., 2016; Buss et al., 2017; but see Jacobi et al., 2017). Corbin et al. (2016) characterized the developmental trajectory for masked word recognition, including testing in the presence of speech-shaped noise. Participants were 5- to 16-year-old children and young adults with normal hearing. As a group, children needed an additional 2.3-dB SNR relative to adults to attain the same correct-response criterion. However, substantial age-related improvements in performance were observed across the age range of children tested. SRTs improved linearly with age until about 10 years of age, but SRTs for older children were indistinguishable from those observed for adults.

## Factors Responsible for Developmental Effects

### Peripheral Encoding

Speech recognition relies on an accurate representation of incoming speech transmitted to the brain *via* the outer ear, middle ear, cochlea, and auditory nerve. Competing noise compromises this representation when the neural excitation produced by target speech and masking noise overlap on the basilar membrane (e.g., Miller, 1947). The term *energetic masking* is often used in the literature to describe the perceptual consequences of degraded peripheral encoding (reviewed by Brungart, 2005). These consequences include reduced audibility, which in turn limits access to acoustic speech features and exerts a negative influence on overall speech intelligibility (e.g., Fletcher and Galt, 1950; Miller and Nicely, 1955).

Extensive research conducted over the past 40 years has focused on understanding the limits of peripheral encoding in children (reviewed by Buss et al., 2012). Results of this work provide converging evidence that school-age children’s speech-in-noise difficulties are not due to immaturity in the sensory representation of speech. Neural transmission through the brainstem auditory pathways appears to be somewhat sluggish during early infancy, but this immaturity appears to resolve by about 6 months of age (e.g., Gorga et al., 1989; Werner et al., 1994). While behavioral data indicate that auditory capabilities related to frequency, intensity, and temporal processing improve during infancy and the early school-age years (Buss et al., 2012), peripheral encoding of the basic properties of sound appears to reach adult-like precision by 6 months of age (reviewed by Eggermont and Moore, 2012). For example, findings from histological, anatomical, and physiological studies indicate mature cochlear function by at least term birth (e.g., Lavigne-Rebillard and Pujol, 1987; Abdala, 2001).

### Listening Strategy

Children’s pronounced speech-in-noise difficulties may be due in part to immature allocation of attention (e.g., Nittrouer et al., 1993; Choi et al., 2008; Youngdahl et al., 2018). Young children show a tendency to listen across a broad range of frequencies, rather than the mature strategy of focusing attention only on regions associated with relevant target speech (e.g., Polka et al., 2008; Youngdahl et al., 2018). In a recent study, Youngdahl et al. (2018) examined whether 5-year-olds, 7-years-olds, or young adults were susceptible to remote-frequency masking in the context of masked sentence recognition. Target sentences were presented in quiet or in noise. Importantly, target speech and masking noise were filtered to ensure no overlap in frequency. Adults and 7-year-olds performed similarly in quiet and masked conditions. In contrast, 5-year-olds performed more poorly in noise than in quiet. These remote-frequency masking effects are in agreement with prior speech detection data reported for infants (Polka et al., 2008), as well as tone-in-noise detection data reported for infants and 4- to 6-year-old children (Bargones and Werner, 1994; Leibold and Neff, 2011).

Children may initially adopt a different listening strategy than adults in order to learn the important speech cues in their native language. This idea is supported by findings from a series of studies conducted by Nittrouer and colleagues investigating the perceptual attention that children and adults assign to the different acoustic components of phonemes (reviewed by Nittrouer, 2002). Whereas preschoolers attend more heavily to speech cues that are dynamic (e.g., formant transitions), adults and children as young as 7 years of age are more influenced by speech cues that are relatively stable across time (e.g., frication noise). This shift in perceptual attention, called the *perceptual weighting shift* (Nittrouer et al., 1993), is consistent with the idea that extensive listening experience is required before mature selective attention abilities emerge.

### Linguistic Knowledge

It has been suggested that children’s pronounced speech-in-noise difficulties reflect their inexperience with language. However, studies that have tested for associations between masked speech recognition and language abilities reveal mixed findings as some studies do not support this association (e.g., Garlock et al., 2001; McCreery and Stelmachowicz, 2011; Nittrouer et al., 2013; Klein et al., 2017; McCreery et al., 2017). Several studies have reported a correlation between children’s speech-in-noise recognition scores and the size of their vocabulary (e.g., McCreery and Stelmachowicz, 2011; Vance and Martindale, 2012), but this relationship has not been observed in other studies (e.g., Eisenberg et al., 2000; Nittrouer et al., 2013).

Discrepancies observed between studies investigating the association between vocabulary knowledge and masked speech recognition may be due to differences in the stimuli used to evaluate this association. Investigators routinely select target speech that falls within the lexicon of the youngest children tested for a given experiment (e.g., Eisenberg et al., 2000; Nittrouer et al., 2013; McCreery et al., 2017). Findings from studies that included later acquired words provide important insight into the association between vocabulary size and masked speech recognition (e.g., Garlock et al., 2001; Klein et al., 2017). Klein et al. (2017) assessed masked word and non-word recognition in a group of 5- to 12-year-old children with hearing loss and an equal number of age-matched children with normal hearing. Vocabulary size for both groups of children was associated with speech-in-noise recognition performance when target stimuli were non-words or later acquired words. In contrast, no association between these two factors was observed when target stimuli were earlier acquired words.

### Working Memory

There has been considerable recent interest in understanding how the cognitive process of working memory influences children’s speech-in-noise recognition abilities. Working memory refers to the temporary storage and processing of incoming sensory information in a memory buffer, allowing for comparisons with stored representations (Baddeley, 2000; Cowan, 2004). Along with speech-in-noise recognition and language skills, working memory abilities improve with age during childhood (e.g., Camos and Barrouillet, 2015).

Data reported in the literature, albeit from a small number of studies, suggest that working memory may play an important role in the development of speech-in-noise recognition. Differences in working memory between children appear to be partly responsible for individual differences in performance on masked speech recognition tests, even when age effects are taken into account (e.g., Magimairaj and Montgomery, 2012; McCreery et al., 2017; but see Magimairaj et al., 2018). McCreery et al. (2017) measured speech-in-noise recognition and performance on four subtests of the Automated Working Memory Assessment (Alloway et al., 2008) in a group of 48 school-age children (5–12 years). Speech recognition was assessed in a speech-shaped noise masker for three types of targets: monosyllabic words, low-predictability sentences, and high-predictability sentences. Children with higher working memory scores showed better speech-in-noise recognition performance for all three types of target stimuli, after controlling for age and vocabulary size.

## Development Of Speech-in-Speech Recognition

Age effects for speech recognition in a masker composed of a small number of speech streams are pronounced relative to those observed in broadband noise with the same long-term average spectrum (e.g., Hall et al., 2002; Wightman and Kistler, 2005; Corbin et al., 2016). For example, Hall et al. (2002) used a forced-choice, picture-pointing task to assess recognition of spondaic words in the presence of speech-shaped noise or two-talker speech. Listeners were 5- to 10-year-old children and 19- to 48-year-old adults. On average, children required an additional 3 dB to perform as well as adults in the noise masker. In contrast, the magnitude of the child/adult difference was 8-dB SNR in the two-talker masker. Larger developmental effects for speech-in-speech relative to speech-in-noise recognition have also been reported using phonemes (Leibold and Buss, 2013), monosyllabic words (e.g., Corbin et al., 2016), and sentences (e.g., Wightman and Kistler, 2005).

Not only are child/adult differences more pronounced for speech-in-speech than for speech-in-noise recognition, mature performance is not reached until the teenage years (e.g., Wightman and Kistler, 2005; Brown et al., 2010; Leibold and Buss, 2013; Corbin et al., 2016). Corbin et al. (2016) assessed children’s (5–16 years) and adults’ word recognition in a two-talker speech masker as well as in a speech-shaped noise masker. Mature SRTs were observed by 10 years of age in the noise masker, but adult-like SRTs for the same children were not observed in the speech masker until after 13 years of age. These observations are consistent with the idea that the factors responsible for developmental effects in speech-in-speech recognition may differ from those responsible for speech-in-noise recognition, and may emerge at different stages of development.

## Factors Responsible for Developmental Effects

### Perceptual Isolation of Target and Masker Speech

The ability to recognize speech produced by one talker when multiple people are talking at the same time relies on central auditory processing. This processing facilitates the grouping of sounds into separate auditory objects and is responsible for the selective allocation of attention (e.g., Bregman, 1990; Bronkhorst, 2000; Best et al., 2007). Collectively, this processing falls within the general framework of *auditory scene analysis* (Bregman, 1990). The perceptual consequences of a failure of grouping and/or selection are sometimes referred to as *perceptual* or *informational masking* (e.g., Carhart et al., 1969; Brungart, 2001). Regardless of terminology, immature grouping and/or selective attention abilities appear to limit the extent to which children perceptually isolate target and masker speech (reviewed by Leibold, 2017).

Auditory grouping refers to the segregation of simultaneous sounds as well as the linkage of sounds over time (e.g., Bregman, 1990; Bronkhorst, 2015). Acoustic differences between target and masker speech influence auditory grouping in adults (e.g., Bregman, 1990; Bronkhorst, 2000; Brungart, 2001; Darwin et al., 2003). For example, speech produced by different talkers tends to vary with respect to multiple acoustic vocal characteristics, including fundamental frequency (F0) and the distribution of formant frequencies (e.g., Fitch and Giedd, 1999). Adults capitalize on these acoustic differences in the context of speech-in-speech recognition, particularly when target and masker speech are produced by talkers that differ in sex (e.g., Festen and Plomp, 1990; Brungart, 2001). Other target/masker acoustic differences that promote auditory grouping and have a positive impact on adults’ speech-in-speech recognition performance include temporal onsets (e.g., Hukin and Darwin, 1995) and binaural cues associated with real or perceived spatial location (e.g., Freyman et al., 2001).

Children appear to take advantage of many of the same acoustic differences between target and masker speech that improve adults’ speech-in-speech recognition performance (e.g., Litovsky, 2005; Cameron et al., 2009, 2011; Yuen and Yuan, 2014; Calandruccio et al., 2016). For example, Litovsky (2005) examined the effect of spatially separating target and masker speech on masked speech recognition performance. Listeners were 4- to 7-year-old children and adults. A forced-choice task with a picture-pointing response was used to estimate SRTs for words embedded in speech-shaped noise, competing sentences produced by one talker, or competing sentences produced by two talkers. Target stimuli were always delivered *via* a loudspeaker positioned directly in front of the listener at 0° azimuth. Maskers were presented from the same location as the target words (co-located) or from a loudspeaker positioned 90° to the side of the listener (separated). Spatial release from masking (SRM) was computed as the difference between the SRTs estimated in the co-located and spatially separated conditions. Children required a more advantageous SNR to achieve the same criterion level of performance as adults in all three masker conditions, but the magnitude of SRM was similar across age. Subsequent studies have confirmed that children benefit from target/masker differences in spatial location in the context of speech-in-speech recognition (e.g., Johnstone and Litovsky, 2006; Cameron et al., 2009; Murphy et al., 2011; Yuen and Yuan, 2014; Corbin et al., 2017). Note, however, that findings from more recent studies indicate that young children experience reduced SRM relative to older children and adults when the target stimuli and/or listening conditions are more challenging (e.g., Cameron et al., 2009; Brown et al., 2010; Yuen and Yuan, 2014; Corbin et al., 2016). For example, Brown et al. (2010) examined sentence recognition in a two-talker masker using the North American Listening in Spatialized Noise-Sentences test (NA LiSN-S). Listeners were a large sample of 12- to 19-year-old children (*n* = 67) and young adults (*n* = 53) with normal hearing. Testing included conditions in which the target and masker were perceived to have originated from the same location in space and conditions in which the target and masker were perceived to be spatially separated. The ability to benefit from perceived spatial separation remained immature until 14 years of age.

Prior studies investigating the extent to which children benefit from acoustic differences between target and masker speech have generally used stimuli that differ across multiple acoustic features (e.g., Litovsky, 2005; Calandruccio et al., 2016; Leibold et al., 2018). For example, Leibold et al. (2018) evaluated whether children and adults benefit from a mismatch in target/masker sex when asked to recognize disyllabic words in a two-talker masker. SRTs for all listeners were higher (i.e., worse) when the target and masker speech were sex matched (e.g., male target speech presented in a male two-talker masker) relative to when target and masker speech were sex mismatched (e.g., male target speech presented in a female two-talker masker). Speech produced by males and females generally differs across multiple acoustic features, including F0, dispersion of formant frequencies, and phonation type (e.g., Fitch and Giedd, 1999). In a later study, Flaherty et al. (2019) observed a striking age effect in the ability to benefit from target/masker differences only in F0, holding other acoustic target/masker differences constant. Whereas adults and older children (>13 years) showed a robust benefit associated with target/masker differences in mean F0, younger children (<7 years) did not. Flaherty et al. (2019) suggested that children might require additional acoustic cues (e.g., distribution of formant frequencies) in order to perceptually isolate target and masker speech. Additional evidence supporting this interpretation is provided by normative data for the LiSN-S clinical test (e.g., Cameron et al., 2009, 2011; Brown et al., 2010). That test battery includes conditions in which the target and masker speech are produced by the same female talker, as well as conditions in which the target and masker speech are produced by different female talkers. While children of all ages tend to show better performance when different talkers produced target and masker speech, adult-like benefit is not observed until 14 years of age.

In addition to auditory grouping, speech-in-speech recognition relies on the ability to selectively attend to the auditory object associated with target speech while disregarding other objects (e.g., Bronkhorst, 2000; Best et al., 2007). Results from several behavioral experiments indicate that children listen less selectively than adults (e.g., Doyle, 1973; Wightman and Kistler, 2005; Leibold and Buss, 2013). For example, Wightman and Kistler (2005) used a dichotic listening paradigm to investigate the influence of selective attention on children’s increased susceptibility to speech-in-speech masking. Listeners were 4- to 16-year-olds and adults. In all conditions, a single target sentence and a single distractor sentence were simultaneously presented to the listener’s right ear. In some conditions, an additional distractor sentence was presented to the listener’s left ear. The task was to repeat back the target sentence while ignoring the distractor sentence(s). Children performed more poorly than adults in all conditions, with developmental improvements observed until about 13 years of age. While the addition of the contralateral distractor sentence negatively impacted performance for listeners of all ages, an analysis of listener error patterns revealed age effects in the ability to disregard speech presented to the contralateral ear. Most errors made by the youngest children tested (4–6 years) were intrusions from the distractor speech presented to the opposite ear as the target sentence. In contrast, errors made by older children and adults were generally intrusions from the distractor speech presented to the same ear as the target sentence.

Despite compelling evidence that selective auditory attention contributes to child/adult differences in masked speech recognition, this area of research remains under-studied. One complicating factor is that the relationship between selective attention and auditory grouping is bidirectional; the formation of auditory objects is influenced by selective attention and vice versa (e.g., Shamma et al., 2011). A related challenge is that we lack behavioral paradigms that can isolate effects of immature selective attention from failures in auditory object formation. Functionally, both processes impact speech-in-speech recognition. Results from electrophysiological studies have provided insight regarding the time course of development of these factors (e.g., Coch et al., 2005; Karns et al., 2015). For example, Karns et al. (2015) examined event-related potentials (ERPs) in the context of a dichotic listening experiment. Listeners were 3- to 5-year-olds, 10-year-olds, 13-year-olds, 16-year-olds, and young adults. Listeners were asked to attend to speech presented to a loudspeaker while ignoring speech presented to another loudspeaker at the same time, or they were asked to attend to speech presented by a male or female talker while ignoring speech produced by a talker that differed in sex. Age-related changes for both tasks were observed in both the latency and morphology of ERPs, with adult-like responses observed only for the oldest two groups of children tested (13 and 16 years).

### Glimpsing

Adults take advantage of brief “glimpses” of target speech available during minima in the envelope of modulated noise (i.e., epochs in which SNR is relatively high), showing better speech recognition performance in modulated or interrupted noise than in nominally steady noise (e.g., Miller and Licklider, 1950; Howard-Jones and Rosen, 1993; Cooke, 2006). Speech maskers composed of a small number of speech streams likewise fluctuate over time. Thus, it has been suggested that children’s increased susceptibility to speech-in-speech masking relative to adults may reflect immaturity in the ability to capitalize on glimpsing opportunities (e.g., Buss et al., 2017; Sobon et al., 2019).

Initial studies investigating children’s speech recognition in temporally modulated noise yielded mixed results regarding child/adult differences in glimpsing (e.g., Stuart, 2008; Hall et al., 2014). More recent studies, however, indicate that school-age children derive less benefit from temporal glimpses in a one- or two-talker speech masker relative to adults (e.g., Buss et al., 2017; Sobon et al., 2019). Buss et al. (2017) evaluated word recognition in a one-talker or a two-talker masker. Listeners were 4- to 16-year-old children and young adults. SRTs were estimated adaptively in each masker, both with and without the addition of a speech-shaped noise. When present, the speech-shaped noise was 10 dB less intense in level than the corresponding speech masker. The rationale for assessing performance with the added noise was to examine the effect of masking the low-level speech cues that would otherwise be available during the envelope minima of the speech masker. The effect of adding noise was larger for older children and adults than for younger children. A follow-up experiment utilized a technique whereby time segments of the combined target and masker speech associated with poor SNRs were removed *via* digital signal processing. The goal of this technique is to approximate ideal segregation of target and masker speech by discarding the time/frequency segments of the stimulus dominated by the masker (e.g., Wang, 2005). Digital segregation reduced the child/adult difference. Nonetheless, young children continued to perform more poorly than older children and adults. Overall, the pattern of results observed across the two experiments reported by Buss et al. (2017) suggests young children are less adept than older children and adults at recognizing speech based on brief glimpses.

Results from Sobon et al. (2019) provide additional evidence that glimpsing abilities limit speech-in-speech recognition during childhood. Speech-in-noise and speech-in-speech recognition were evaluated in 8- to 10-year-olds and young adults. Data were collected using an adaptive sentence recognition task and subsequently fitted with psychometric functions. Similar psychometric slopes were observed for children and adults in the speech-shaped noise masker, but slopes were steeper for children than for adults in the two-talker masker. This result was interpreted as indicating that children were not able to benefit from transient improvements in SNR in the two-talker masker to the same extent as adults. This interpretation received additional support from an analysis using the extended speech intelligibility index (Rhebergen and Versfeld, 2005), to estimate the audibility of speech cues required for recognition. Children required more audibility overall than adults, but this difference was larger for the two-talker masker than the speech-shaped noise masker. These results are consistent with the idea that children’s immature speech-in-speech recognition is at least partly due to reduced glimpsing abilities. Immature segregation, selective attention, or a combination of these two effects may contribute to young children’s reduced ability to recognize speech based on sparse cues.

## Summary and Future Directions

Data summarized in this review provide compelling evidence that the ability to recognize masked speech follows a prolonged time course of development. Children have more difficulty recognizing speech in the presence of background sounds relative to adults, with age effects reported for a wide range of stimuli and listening conditions. Research on children’s speech recognition in steady-state noise indicates that child/adult differences persist until about 9–10 years of age (e.g., McCreery and Stelmachowicz, 2011; Corbin et al., 2016). In contrast, child/adult differences appear to be larger and extend into adolescence when the masker is also speech (e.g., Hall et al., 2002; Brown et al., 2010; Corbin et al., 2016; Buss et al., 2017; but see Dillon et al., 2018). Masker-dependent differences in the time course of development highlight the importance of incorporating both listener and stimulus factors into models of masked speech recognition.

A focus for this review was to consolidate what is known about the factors responsible for developmental effects in masked speech recognition. Recognizing speech in the presence of background sounds depends upon on multiple stages of auditory, cognitive, and linguistic processing. It is important to highlight that immature processing within any stage of processing is likely to influence the extent to which children hear and understand speech in their everyday lives. It is well established that degradations in peripheral encoding negatively influence speech recognition (e.g., Miller and Nicely, 1955), but is perhaps less obvious to researchers outside the field that an immature ability to perceptually isolate target and masker speech can result in the same functional consequences. Efforts are needed to establish models that account for maturational effects, taking into account the specific contributions of the multiple factors and processes required to recognize masked speech.

There are a number of key challenges to address in future research. Efforts are underway to understand the many factors that affect children’s masked speech recognition abilities, including age, audibility, masker complexity, working memory, and language skills (e.g., Lang et al., 2017). Another long-standing issue is the general dearth of behavioral paradigms and psychometric methods required to understand and quantify contributions of auditory grouping, selective attention, and/or more general cue requirements to children’s speech-in-speech recognition abilities. As recent data by Sobon et al. (2019) indicate, factors such as the slope of the psychometric function and the SNR at which a criterion threshold is reached can provide more accurate and detailed estimates of child/adult differences than the conventional approach of considering threshold data alone. Finally, the studies discussed in this review involved children with typical development. Future research is needed to determine how listener factors such as peripheral hearing loss, neurological abnormalities, limited language experience, and cognitive impairment impact children’s masked speech recognition abilities (e.g., Hillock-Dunn et al., 2015; Chermak et al., 2017).

## Author Contributions

LL and EB both contributed to the writing of the review.

### Conflict of Interest Statement

The authors declare that the research was conducted in the absence of any commercial or financial relationships that could be construed as a potential conflict of interest.
